# Pan-cancer analysis shows that TRIP13 as a potential prognostic and immunotherapeutic biomarker for multiple cancer types including LIHC and LUAD

**DOI:** 10.1097/MD.0000000000042588

**Published:** 2025-05-30

**Authors:** Chong Li, Ziyu Zhou, Yi Liu, Yao Guo, Tingjun Liu, Jie Teng, Piao Chen, Ankang Hu, Lianlian Wu, Dandan Qiao, Quangang Chen, Jing Liu

**Affiliations:** a Graduate School, Xuzhou Medical University, Xuzhou, Jiangsu Province, China; b Center of Animal Laboratory, Xuzhou Medical University, Xuzhou, Jiangsu Province, China; c Department of Cell Biology and Neurobiology, Xuzhou, Jiangsu Province, China; d Department of Respiratory Medicine, Xuzhou Central Hospital, Xuzhou, Jiangsu Province, China.

**Keywords:** immune cell infiltration, LIHC, Pan-cancer, prognosis, TRIP13

## Abstract

Growing evidence indicates that thyroid hormone receptor interactor 13 (TRIP13) plays an oncogenic role in various malignancies. Pan-cancer analysis was performed to elucidate the prognostic value and oncogenic role of TRIP13, and detect TRIP13 expression levels in diverse cancer types. We found that TRIP13 was overexpressed in multiple tumors. Subsequently, we explored the relationship between TRIP13 expression-associated alterations and clinical prognosis, DNA methylation, immune cell infiltration, immune checkpoints, tumor mutational burden, microsatellite instability, and drug sensitivity. Gene set enrichment analysis was utilized to explore the molecular mechanism of TRIP13. A nomogram was developed to predict the impact of TRIP13 expression on an liver hepatocellular carcinoma prognosis. The miRDB database was used to predict miRNA targets for TRIP13. The results indicated that TRIP13 overexpression was associated with a poor overall survival, disease-specific survival, and progression-free interval in various cancer types. The mutation frequency of TRIP13 was the highest in individuals with lung squamous cell carcinoma. TRIP13 expression levels were negatively associated with immunocyte infiltration, immune scores, tumor mutational burden, microsatellite instability, and DNA methylation in certain cancers, and positively correlated with the expression of at least 5 immune checkpoint-related genes in bladder carcinoma, breast cancer, kidney clear cell carcinoma, liver hepatocellular carcinoma, lung adenocarcinoma, mesothelioma, and thyroid cancer. In addition, we discovered that TRIP13 participated in the cell cycle during Gene Ontology and Kyoto Encyclopedia of Genes and Genomes enrichment analysis for most cancers. The areas under the curve for the 1-, 3-, and 5-year survival rates in the nomogram exceeded 0.6, indicating that the model has good predictive performance, and was validated using samples obtained from liver hepatocellular carcinoma patients. We also found that miR-656-3p tends to bind to TRIP13 mRNA and regulate TRIP13 expression. Additionally, we identified 26 drugs sensitive to tumors with high TRIP13 expression levels from the CTRP and GDSC databases. Finally, the promoting effect of TRIP13 on lung cancer was verified, the results demonstrating TRIP13 could accelerate lung cancer cell proliferation, migration, and invasion. Collectively, our findings suggest that TRIP13 may act as a potential prognostic marker and novel target for cancer therapy.

## 1. Introduction

The International Agency for Research on Cancer reported that the incidence rate of cancer in individuals under the age of 50 has been rising rapidly worldwide since 1990. One in 5 males or females will develop cancer in their lifetime. Despite advances in healthcare significantly extending lifespans, cancer remains a significant contributor to global mortality. Approximately 1 in 9 males and 1 in 12 females will die from cancer.^[1]^ Given the aging population, the number of cancer-related deaths is expected to continue to rise, resulting in a greater public health burden. However, the mechanisms of cancer occurrence are highly complex, rendering the pan-cancer analysis of genes essential. Assessing their relationship with clinical prognosis and exploring potential molecular mechanisms are of significant scientific value and clinical importance.

Thyroid hormone receptor interaction factor 13 (TRIP13) encodes a member of the highly conserved AAA-ATPase family that contributes to cancer susceptibility. Previous studies have shown that TRIP13 is involved in the regulation of spindle assembly checkpoint signaling and DNA damage repair during cell division,^[2]^ and cells lacking TRIP13 cannot activate the spindle assembly checkpoint.^[3]^ Therefore, abnormal TRIP13 expression levels in cancer cells can lead to errors in chromosomal separation. This may result in chromosomal instability and serve as a tumor susceptibility locus.^[2]^ Several studies revealed that TRIP13 can drive tumorigenesis and promote tumor progress, leading to a poor prognosis for individuals with prostate cancer,^[4]^ bladder cancer,^[5]^ B-cell lymphoma, and multiple myeloma.^[6]^ A thorough examination of the role of TRIP13 in tumor occurrence and progression, along with an investigation of its potential carcinogenic mechanisms, is crucial for identifying potential therapeutic targets for various cancers.

In the current study, we conducted a pan-cancer analysis of TRIP13 using data available online. We have systematically discussed the association between the TRIP13 expression level and clinical prognosis, tumor microenvironment (TME), DNA methylation, tumor mutational burden (TMB), microsatellite instability (MSI), and drug sensitivity in 33 cancers. Gene set enrichment analysis was performed through Kyoto Encyclopedia of Genes and Genomes (KEGG) and Gene Ontology (GO) analysis. In addition, a nomogram for predicting 1-, 3-, and 5-year overall survival (OS), disease-specific survival (DSS), and progression-free interval (PFI) in LIHC patients was developed and validated using the TCGA-LIHC cohort. Finally, the miRNA target to *TRIP13* was predicated by miRDB and miRanda algorithm using online database.

## 2. Methods

### 2.1. Gene expression analysis

The Gene_DE module in TIMER2.0 (https://timer.cistrome.org) was utilized to determine the expression profile of TRIP13 in various cancers, and compare tumor and healthy tissues. The UALCAN portal, accessible at https://ualcan.path.uab.edu/analysis-prot.html, is an interactive online tool that enables the study of cancer Omics data. We utilized this resource to perform protein expression analysis on the CPTAC dataset.

### 2.2. Survival prognosis analysis

The TCGA database has been used to extract survival and clinical data for different types of cancers. Our study investigated the relationship between TRIP13 expression levels and the prognosis of individuals with 33 different types of cancer. We used forest plots within Kaplan–Meier curves in the Kaplan–Meier Plotter (https://kmplot.com/analysis/), UCSC Xena Shiny (https://xenabrowser.net/datapages/), and GEPIA2 (https://gepia2.cancer-pku.cn/#index) to analyze these associations. Survival studies were conducted using Kaplan–Meier and forest plot curves.

TCGA and GTEx data sets were obtained, and a receiver operating characteristic (ROC) curve was constructed using the Survival ROC software tool.

The horizontal and vertical axes of the ROC curve graph corresponded to the false positive and true positive rates, respectively. A larger area under the ROC curve (AUC) indicates improved accuracy in predicting the prognosis.

### 2.3. Genetic mutation analysis

cBioPortal is a freely accessible online platform (https://www.cbioportal.org/) designed for the exploration, visualization, and analysis of complex cancer genomics data. The website provides information on the genetic changes in TRIP13, including the types of mutations, copy number alterations, and the frequency of these variations in each TCGA cancer type. Information on the specific location of the mutations was also made available.

In this study, the genetic mutation data for 33 distinct cancer types were obtained from a database maintained by TCGA. The TMB of each sample was determined using the Perl programming language. The “grader” R package was utilized to generate radar plots, whereas Spearman correlation analysis was employed to examine the association between the differential expression of TRIP13 protein, TMB, and MSI.

The statistical significance between disparities in TRIP13 methylation levels between healthy and tumor tissues was assessed using the Wilcoxon signed-rank test and outcomes were graphically shown using ggplot2 R software.

### 2.4. Exploration of the TME landscape

We utilized the “Immune-Gene” module of the TIMER2.0 web server to examine the correlation between TRIP13 expression and immune infiltration in all TCGA cancers. The Spearman rank correlation test, corrected for purity, was utilized to get the *P*-values and partial correlation (cor) values. Data were shown using a heatmap and a scatter plot. The R packages “violet,” “ggplot2,” and “ggpubr” were utilized to create boxplots and a heatmap was used to examine the Spearman correlation coefficients between gene expression levels and several immune-related molecules.

### 2.5. *TRIP13*-associated gene enrichment analysis

The PPI networks of the top 20 TRIP13-binding proteins were obtained using the STRING website (version 11.0b; https://string-db.org/). The GeneMANIA database was utilized to produce a list of the top 20 associated genes. The database can be accessed at https://genemania.org/.

The Limma R software package, specifically version 3.40.2, was utilized to assess variations in mRNA expression levels. The screening parameters for differential mRNA expression were set at a significance level of *P* < .05 and an absolute fold change >1. GOKEGG analyses facilitated the examination and comparison of distinct signaling pathways and biological effects between LIHC tumor and healthy tissue samples. The “clusterProfiler” tool in R software facilitated the assessment of GO and KEGG pathways. Items with *P* < .05 and Q < 0.05 were considered to be statistically significant. This significance was visually indicated using bubble and circle plots.

### 2.6. Gene set enrichment analysis (GSEA)

TCGA data were classified into 2 groups based on the differential expression of TRIP13 genes. Differentially expressed genes linked with TRIP13 were then retrieved. The R packages “limma (version 3.44.3),” “org.Hs.e.g..db (version 3.11.4),” “clusterProfiler (version 3.16.1),” and “enrichplot (version 1.8.1)” were employed for functional analysis.

### 2.7. Construction and evaluation of nomogram

We created a predictive diagram using the TRIP13 expression levels and the OS, DSS, and PFI. To validate the predicted nomogram accuracy, we created calibration curves for the 1-, 3-, and 5-year survival rates. In addition, we created decision curve analysis curves for 1-, 3-, and 5-year periods to evaluate the advantages provided by the nomogram.

### 2.8. Drug resistance analyses

Gene expression data and pharmacological data were obtained from the Genomics of Drug Sensitivity in Cancer (GDSC, https://www.cancerrxgene.org/) (Iorio et al, 2016) and Cancer Therapeutics Response Portal (CTRP, https://portals.broadinstitute.org/ctrp/) (Basu et al, 2013) and analyzed to investigate the relationship between TRIP13 and drug resistance.

### 2.9. Prediction of miRNA targets of TRIP13

Predicted target genes of TRIP13 were determined using the miRDB (https://www.mirdb.org/) and miRanda (https://www.mirbase.org/) databases. To enhance the prediction precision, we chose target genes that were able to identify 7 specific miRNAs.

### 2.10. Cell culture and transfection

A549 cells were cultured in DMEM containing 10% fetal bovine serum (Sunrise, China), and 1% penicillina/streptomycin. A lentiviral vector overexpressing TRIP13 was constructed and transduced into A549 cells, thereby generating a stable cell line with TRIP13 overexpression. In parallel, a specific siRNA targeting TRIP13 was designed and transfected into A549 cells, aiming to effectively interfere TRIP13 expression.

### 2.11. Cell vialility assays

TRIP13 was overexpressed or knockdown in A549 cells, and cells were seeded into 96-well plates at a density of 5 × 10³ cells per well. The cells were then incubated for 0, 24, 48, or 72 hours. After discarding the culture medium, a mixture of 90 μL culture medium and 10 μL CCK-8 reagent (VICMED, VC5001L-2500) was prepared in a 1.5 mL EP tube and added to each well. The cells were incubated at 37 °C for 1 hour, and absorbance was measured at 450 nm using a microplate reader to assess cell viability.

### 2.12. Western blot

After the A549 cells were subjected to a series of treatments, they were lysed using RIPA buffer. The lysates were then centrifuged at 12,000 × *g* for 15 minutes at 4 °C to pellet the cellular debris. The supernatant containing the soluble proteins was carefully collected. Equal amounts of protein were then loaded onto a 10% SDS-polyacrylamide gel for electrophoresis. After that, the proteins were transferred onto a polyvinylidene difluoride membrane. The membrane was then blocked with 5% nonfat milk for 1 hour at room temperature. The membrane was subsequently incubated with the primary antibody against TRIP13 (A17357, Abclone, China) and β-actin (AC038, Abclone, China) overnight at 4 °C. After washing with TBST, the membrane was incubated with the corresponding secondary antibody conjugated to horseradish peroxidase for 1 hour at room temperature. The protein bands were visualized using an enhanced chemiluminescence detection system.

### 2.13. Transwell assay

The A549 cells were cultured in serum-free medium in the upper chamber and 800 μL of complete medium was added to the lower chamber. The cultures were incubated at 37 °C for 24 or 48 hours. After incubation, cells that migrated to the underside of the membrane were stained and observed by microscope.

### 2.14. Wound healing assay

The A549 cells were inoculated in 6-well plates and allowed to grow until fusion reached 100%. A 200 μL sterile pipette tip was used to form a scratch on the monolayer. The medium was then discarded and the wells were washed with PBS to remove isolated cells. Each well was then supplemented with 2 mL of serum-free incomplete medium and incubated for 24 or 48 hours, and observed by microscope.

### 2.15. Statistical analysis

We performed statistical analyses using R (version 4.1.3) and GraphPad Prism 8. Logistic regression analysis was conducted using SPSS (version 26.0). Two groups were analyzed using the Student *t* test, and more than 2 groups were analyzed with a one-way analysis of variance. *P* < .05 indicates statistical significance, and all the analyses were performed according to the two-sided tests (*P < .05, **P < .01, ***P < .001, “–”: not significant).

## 3. Results

### 3.1. Pan-cancer expression of TRIP13

**Figure 1.**
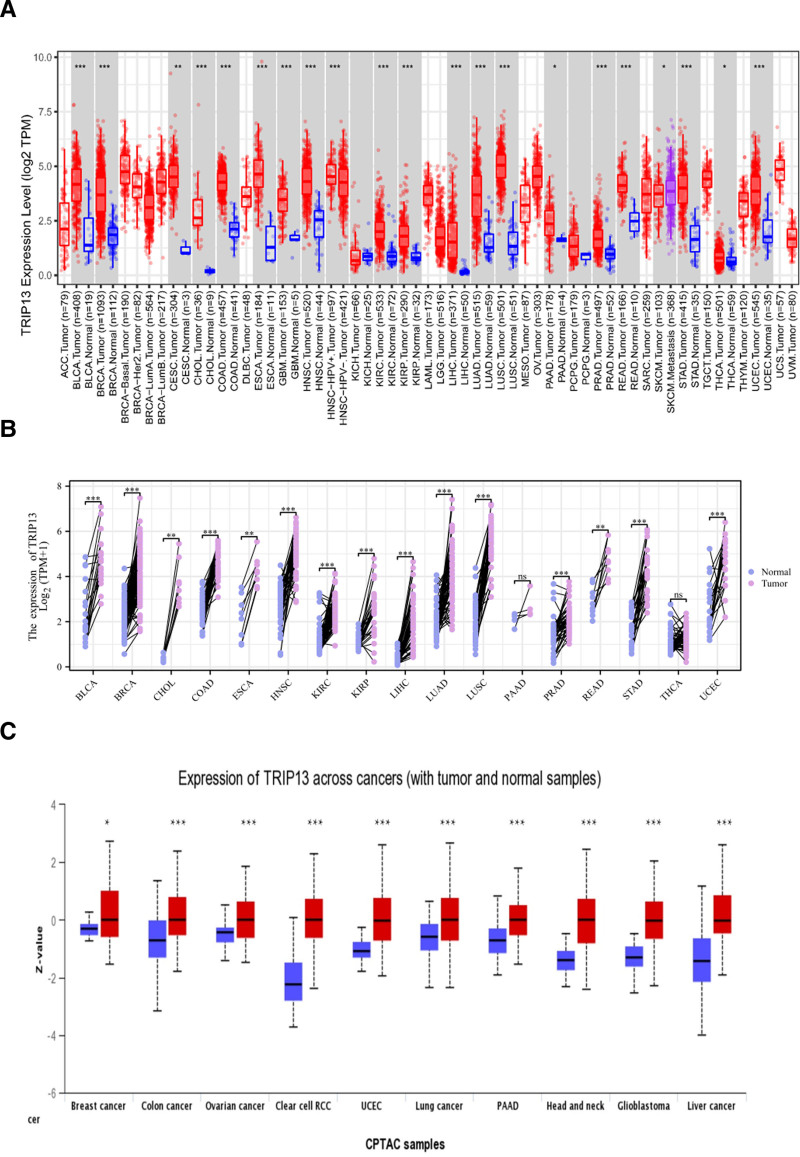
The expression level of TRIP13 in different tumor tissues and corresponding adjacent healthy tissues. (A) The expression status of TRIP13 in different cancers or specific cancer subtypes was analyzed through TIMER 2.0. (B) The differential expression of TRIP13 was analyzed in paired samples of 17 types of tumors. (C) Based on the CPTAC dataset, we also analyzed the expression level of TRIP13 protein between healthy tissues and primary BRCA, OV, COAD, ccRCC, UCEC, LUND, PAAD, HNSC, GBM, and LIHC tissues using the UALCAN dataset. *P < .05; **P < .01; ***P < .001.

The TIMER2.0 program was utilized to analyze variations in TRIP13 expression in various cancer types within the TCGA database. Figure 1A shows that the expression levels of TRIP13 in tumor tissues of various cancer types, including cholangiocarcinoma (CHOL), colon adenocarcinoma (COAD), cervical squamous cell carcinoma, glioblastoma multiforme (GBM), lung squamous cell carcinoma (LUSC), esophageal carcinoma (ESCA), stomach adenocarcinoma (STAD), head and neck squamous cell carcinoma (HNSC), rectum adenocarcinoma (READ), lung adenocarcinoma (LUAD), bladder urothelial carcinoma (BLCA), breast invasive carcinoma (BRCA), kidney clear cell carcinoma (KIRC), kidney renal papillary cell carcinoma (KIRP), liver hepatocellular carcinoma (LIHC), pancreatic adenocarcinoma (PAAD), prostate adenocarcinoma (PRAD), thyroid carcinoma (THCA), and uterine corpus endometrioid carcinoma (UCEC), are higher than those in the corresponding control tissues.

In this study, a comparison was conducted of the expression of TRIP13 in matched samples and 17 tumor tissues, encompassing various cancer types, such as BLCA, BRCA, CHOL, COAD, ESCA, HNSC, KIRC, KIRP, LIHC, LUAD, LUSC, PAAD, PRAD, READ, STAD, THCA, and UCEC. Additional examinations indicated that except for PAAD and THCA, TRIP13 expression levels in tumor tissue samples were considerably higher than those in healthy tissues (*P* < .01) (Fig. 1B). These findings support the results depicted in Figure 1A.

We also investigated the differences in TRIP13 expression between healthy and tumor tissues for ovarian serous cystadenocarcinoma (OV), clear cell RCC (RCC), BRAC, COAD, LUSC, PAAD, HNSC, GBM, UCEC, and LIHC using the UALCAN dataset. The results were consistent with those obtained in previous studies (Fig. 1C).

### 3.2. The potential connection between the differential expression of TRIP13 and pan-cancer prognosis

**Figure 2.**
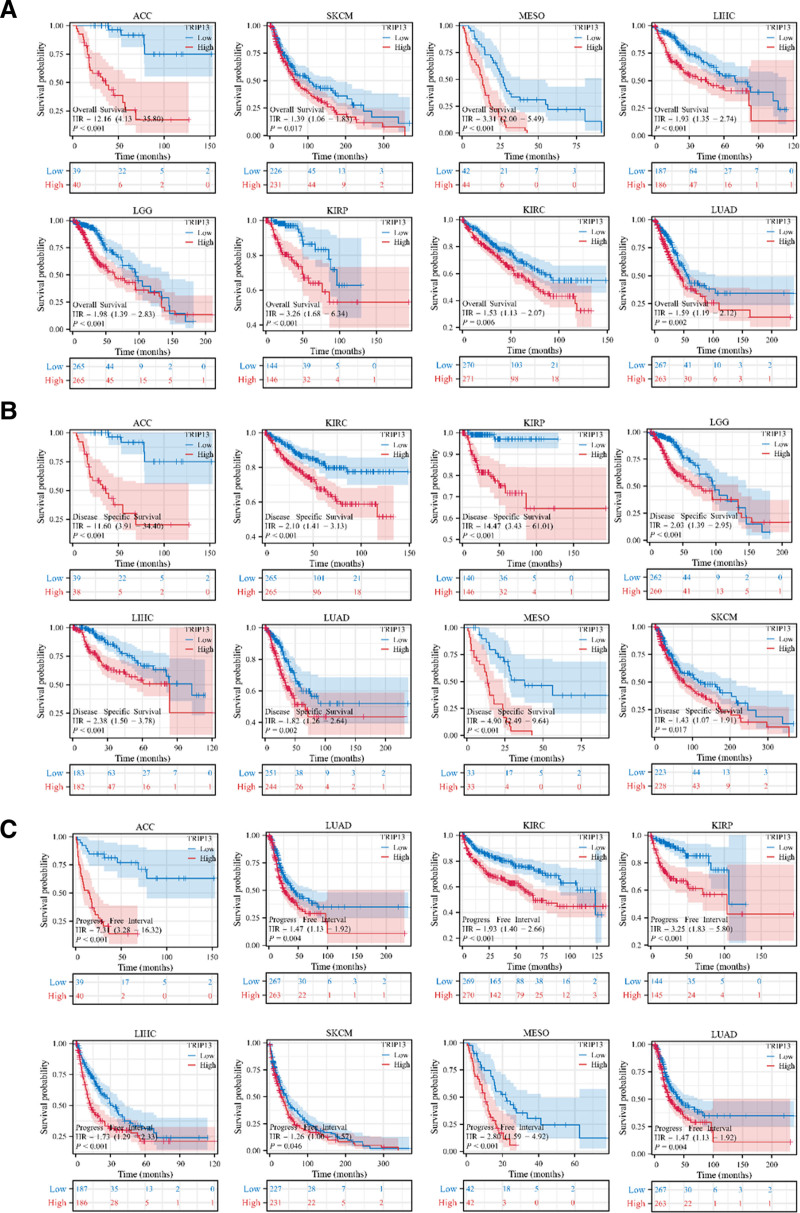
Correlation between TRIP13 gene expression levels and pan-cancer survival prognosis. Survival curves showing the relationship between TRIP13 expression and OS (A), DSS (B), and PFI (C) in pan-cancer (*P* < .05).

Kaplan–Meier survival analysis was performed to investigate the association between clinical outcomes and TRIP13 expression across various cancer types, to detect the effect of TRIP13 on OS, DSS, and PFI. As shown in Figure 2A, increased TRIP13 expression was associated with a shorter OS in the following forms of cancer: KIRP, LIHC, ACC, skin cutaneous melanoma (SKCM), mesothelioma (MESO), LUAD, KIRC, and brain lower-grade glioma (LGG). In addition, during DSS data analysis (Fig. 2B), we found an inverse correlation between TRIP13 expression and prognosis in patients diagnosed with ACC, KIRC, KIRP, LGG, MESO, LIHC, SKCM, and LUAD. To examine the association between TRIP13 expression and PFI (Fig. 2C), we explored the potential negative impact of elevated TRIP13 expression on the PFI of patients with ACC, KIRC, KIRP, LIHC, MESO, LUAD, LGG, and SKCM.

**Figure 3.**
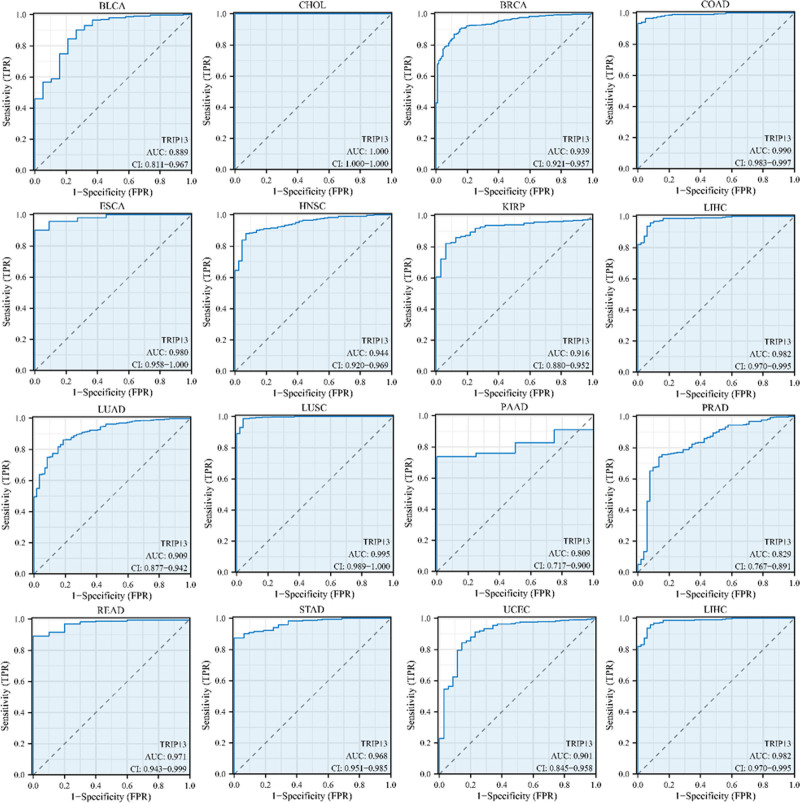
Analysis of receiver operating characteristics (ROC) curves between TRIP13 and tumor prognosis based on data from the TCGA and GTEx databases.

Figure 3 demonstrates that TRIP13 can serve as a biomarker with exceptional sensitivity and specificity for diagnosing different forms of cancer. The area under the curve (AUC) values for the following cancer types exceeded 0.8: BLCA (AUC = 0.889), CHOL (AUC = 1.000), BRCA (AUC = 0.939), COAD (AUC = 0.990), ESCA (AUC = 0.980), HNSC (AUC = 0.944), KIRP (AUC = 0.916), LIHC (AUC = 0.982), LUAD (AUC = 0.909), LUSC (AUC = 0.995), PAAD (AUC = 0.809), PRAD (AUC = 0.829), READ (AUC = 0.971), STAD (AUC = 0.968), and UCEC (AUC = 0.901).

### 3.3. Landscape of TRIP13 mutation profiles in different tissues

**Figure 4.**
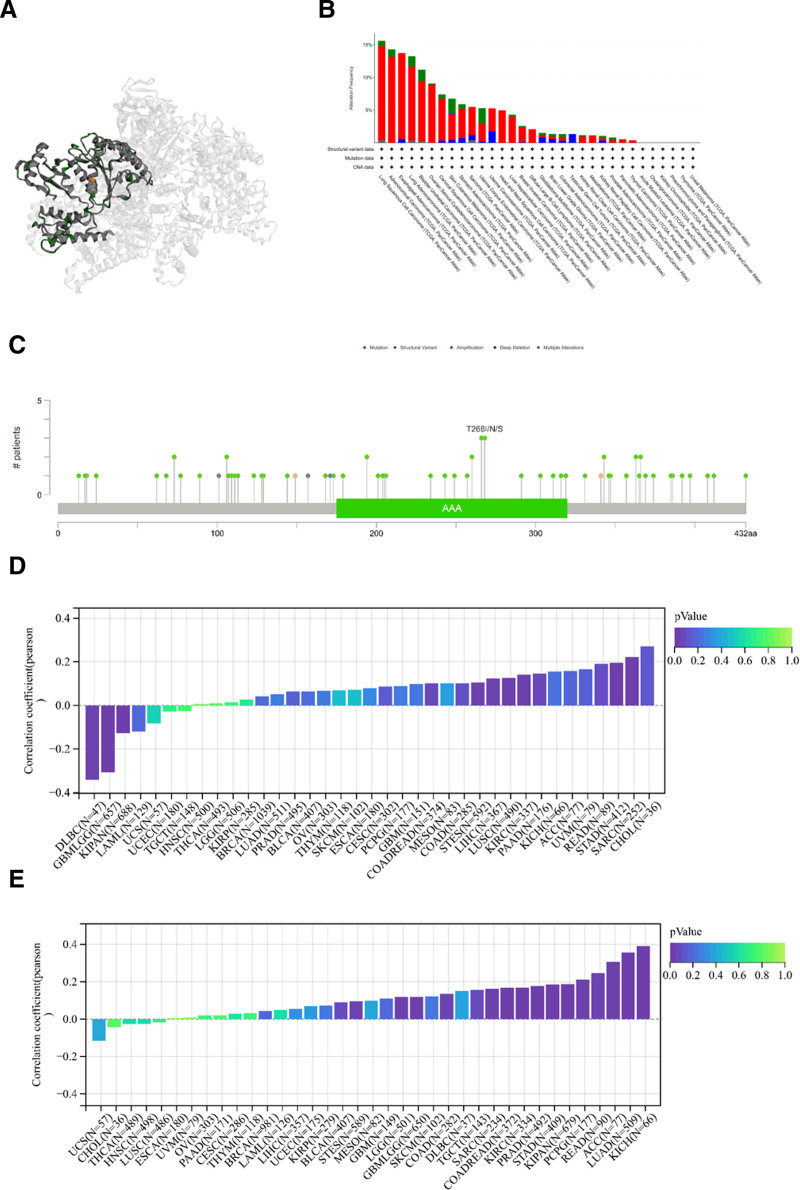
Genetic alterations in TRIP13 in various tumor types. The mutation associated features of *TRIP13* in TCGA tumors were analyzed using the cBioPortal tool. (A) The 3D structure of TRIP13 is displayed. (B) The alteration frequency along with mutation type is displayed. (C) Mutation sites are displayed. (D) Correlation between TRIP13 expression levels and MSI. (E) Correlation between TRIP13 expression and TMB.

We utilized the cBioportal database to analyze the mutation status of TRIP13 and generate a three-dimensional representation of the protein (Fig. 4A). Based on the findings, the “amplification” alteration was observed most commonly, accounting for 14.37% of alterations. Additionally, *TRIP13* had the highest alteration frequency, specifically in patients with LUSC (Fig. 4B). In addition to LUSC, mutations in *TRIP13* are highly prevalent in ACC, ESCA, LUAD, and BLCA, with rates above 10%. Figure 4C displays comprehensive details regarding the forms, locations, and numbers of *TRIP3* mutations. The most common mutation sites are A266V/S, T268I/N/S, Q194H, I343M/V, E363K/V, V366M, G106S/V, and V73A/L.

TMB represents the number of DNA base mutations per million tumor samples. Deletions or insertions of repeating units in tumor tissue compared to healthy tissue result in the generation of novel microsatellite alleles, leading to alterations in microsatellite length in MSI. MSI and TMB are associated with an elevated susceptibility to cancer. We examined the correlation between TMB (tumor mutational burden) and MSI (microsatellite instability) as well as TPIP3 expression levels across various forms of cancer. As shown in Figure 4D, a statistically significant positive correlation was observed between TRPI3 expression and MSI in COAD, READ, STES, LIHC, LUSC, KIRC, STAD, and SARC, but a negative correlation was observed with DLBC, GNMLGG, and KIPAN. Furthermore, in patients with BLCA, STES, LGG, glioma (GBMLGG), COAD, TGCT, SARC, COAD, READ, KIRC, PRAD, STAD, KIPAN, pheochromocytoma and paraganglioma (PCPG), READ, ACC, LUAD, and KICH, the overexpression of TRIP13 was positively correlated with TMB (Fig. 4E).

### 3.4. Pan-cancer analysis of TRIP13 methylation

**Figure 5.**
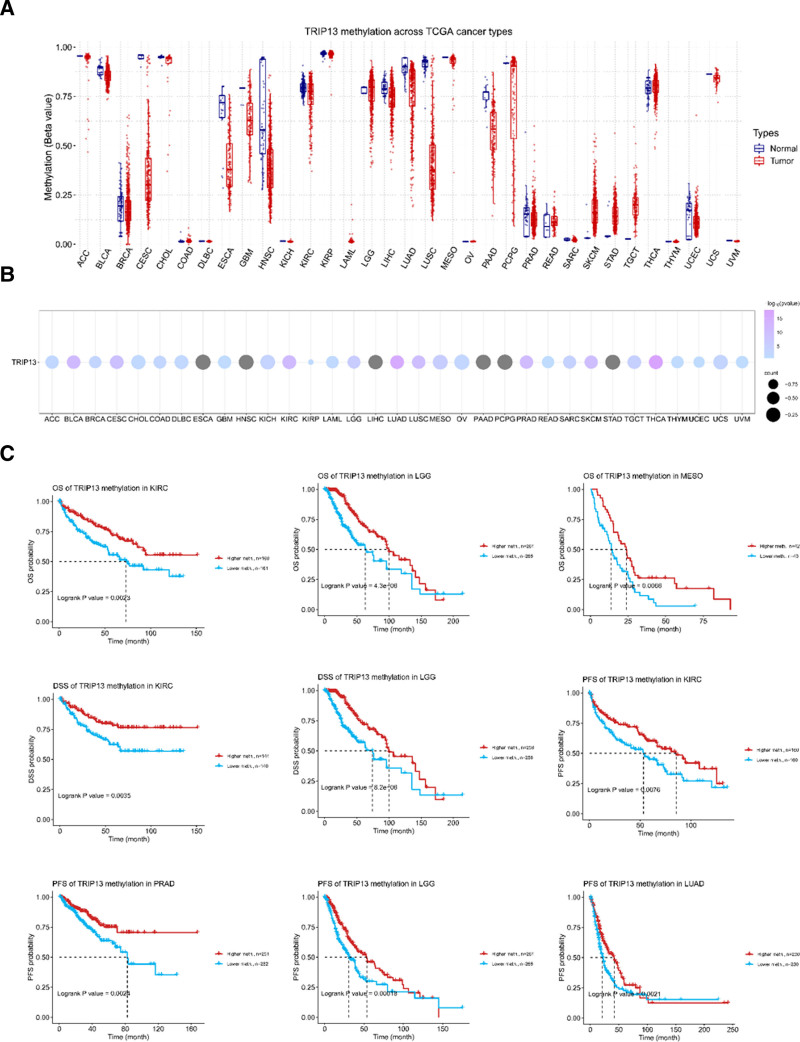
Differences in TRIP13 methylation levels and their prognostic value. (A) Differences in the methylation (β-value) levels of TRIP13 in healthy and tumor tissues of 33 tumor types. (B) Correlation of TRIP13 expression with methylation. (C) Kaplan–Meier analysis of OS with TRIP13 methylation levels in KIRC, LGG, and MESO.

DNA methylation plays a role in regulating gene expression and cancer progression. Therefore, the DNA methylation levels of *TRIP13* in human cancer were investigated using the GCSA database. The findings demonstrated a robust correlation between the DNA methylation levels of BLCA, BRCA, ESCA, HNSC, KIRC, LIHC, LUAD, LUSC, PAAD, PRAD, THCA, and UCEC and TRIP13expression. A strong negative association was observed between TRIP13 expression and tumor samples from patients with BLCA, BRCA, ESCA, HNSC, KIRC, LIHC, LUAD, LUSC, PAAD, PRAD, and UCEC (Fig. 5A and B). Conversely, a strong positive correlation was discovered between TRIP13 expression and THCA tumor samples. Moreover, this study investigated the correlation between TRIP13 methylation levels and cancer prognosis. The study involved the categorization of individuals with various tumor types into 2 groups based on whether they had high or low levels of TRIP13 methylation. Our findings indicate that patients with KIRC and LGG, who had a longer DSS, showed higher levels of TRIP13 methylation. In addition, the TRIP13 high-methylation group, which included patients with KIRC, LGG, and MESO, exhibited an extended OS. Patients with KIRC, PRAD, LGG, and LUAD, who exhibited an extended progression-free survival (PFS), were likely to exhibit high levels of TRIP13 DNA methylation, as shown in Figure 5C.

### 3.5. Correlation among TRIP13 expression levels and tumor immune cell infiltration

**Figure 6.**
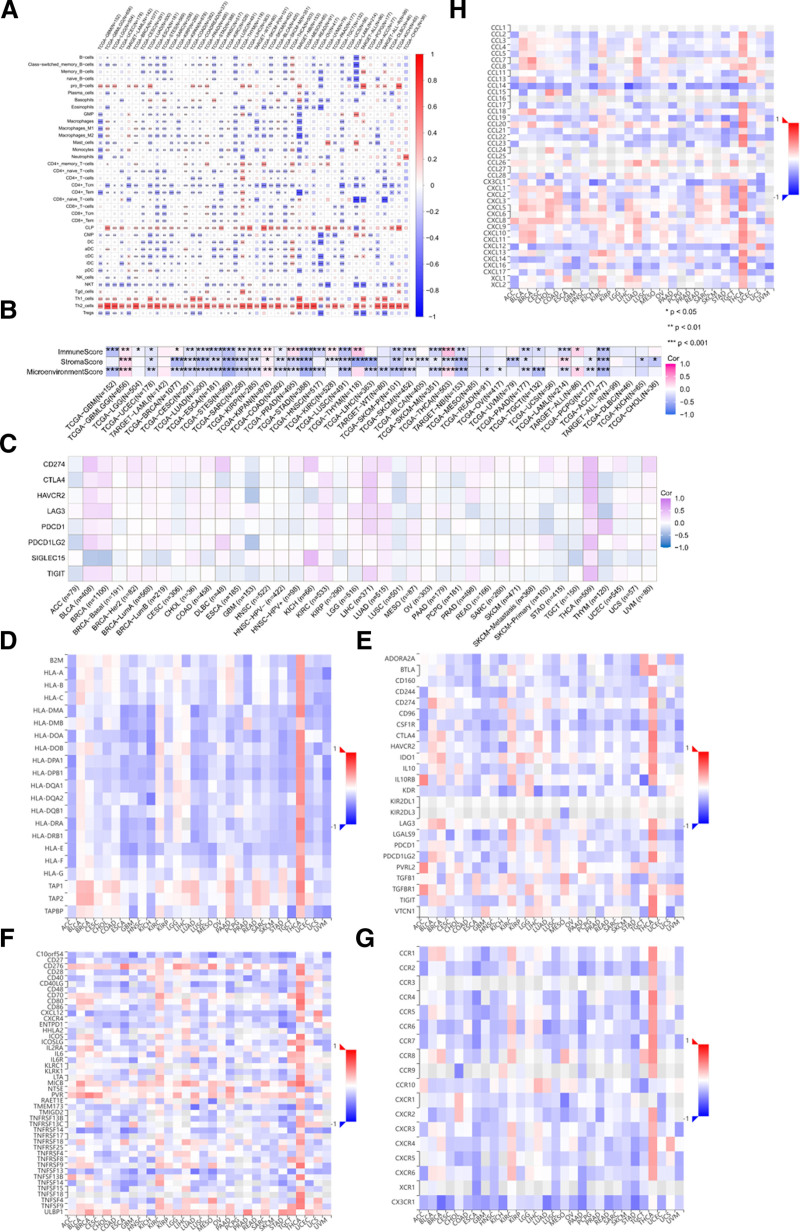
Effects of TRIP13 on the tumor immune microenvironment. (A) Examining the relationship between TRIP13 expression and immune infiltration using the TIMER 2.0 database and xCELL database (B) *P < .05; **P < .01; ***P < .001. The TRIP13 expression levels were correlated with the immune checkpoints (C), chemokines (D), chemokine receptors (E), immunostimulatory factors (F), immunosuppressive factors (G), and MHC molecules (H); the red color represents a positive correlation and blue color represents a negative correlation.

The presence of immune cells is crucial for cancer progression. Hence, we examined the relationship between TRIP13 expression and immune cell infiltration in 43 forms of cancer using the TIMER2.0 database (Fig. 6A) and the xCell database (Fig. 6B). We observed a notable positive correlation (|correlation coefficient|>0.3, *P* < .05) between TRIP13 levels and Th2 cell infiltration in all malignancies except for OV. However, in most tumors, a negative association was observed between TRIP13 expression levels and immune cell infiltration. Conversely, the presence of Th1 cells was inversely related to the expression of TRIP13 in patients with THCA, SKCM, and UVM. However, there is a substantial positive association between the presence of Th1 cells and TRIP13 expression levels in GBM, GBMLGG, BRCA, LUAD, STES, STAD, BLCA, and neuroblastoma (NB) (|correlation coefficient|>0.3, *P* < .05). In addition, the xCell database was used to analyze the relationship between TRIP13 expression and the stromal score, immunological score, and microenvironment score for these 43 types of cancer. The results revealed a high positive correlation between TRIP13 expression and immune scores in THCA, but a significant negative correlation with immune scores in GBM, STES, STAD, LUSC, and ACC. Currently, there is a significant inverse correlation between TRIP13 expression and the stromal scores of ACC, NB, LUAD, STES, PRAD, STAD, LUSC, and LIHC. The microenvironmental scores of STES, STAD, NB, TGCT, and ACC exhibited a notable inverse association with TRIP13 expression levels, while the microenvironmental scores of THCA had a significant positive correlation (|correlation coefficient|>0.3, *P* < .05). A recent meta-analysis of 442 individuals with different types of solid tumors revealed a robust association between myeloid-derived suppressor cells (MDSC) and reduced OS, disease-free survival, and PFS. Furthermore, studies have shown that myeloid-derived immunosuppressive cells (PMN-MDSCs) within the tumor microenvironment are responsible for oxidative lipid production, self-ferroptosis, and T-cell activity suppression. This study focused on investigating the effect of TRIP13 on the extent of MDSC infiltration in various cancer types using the TIMER2.0 database (Figure S1, Supplemental Digital Content, https://links.lww.com/MD/P87). TRIP13 expression and MDSC invasion were significantly correlated in all 40 malignancies or cancer subtypes in the TIMER2.0 database, except for THCA and UCS.

Next, the relationship between immune-associated genes and differential TRIP13 expression was investigated in 33 distinct tumor types. The genes encoded proteins such as immune checkpoints, chemokines, chemokine receptors, immunostimulatory factors, immunosuppressive factors, and MHC molecules (Fig. 6C–H). We observed a positive association between the expression of TRIP13 and at least 5 genes related to immunological checkpoints in BLCA, BRCA, KIRC, LIHC, LUAD, MESO, and THCA. In addition, the expression of TRIP13 showed a negative correlation with a majority of immune-related molecules, encompassing 41 chemokines, 18 chemokine receptors, 45 immunostimulants, 24 immunosuppressants, and 21 important MHC components. These findings suggest that TRIP13 has a crucial function in controlling the immune response to human cancer.

### 3.6. Enrichment of TRIP13-associated partners

**Figure 7.**
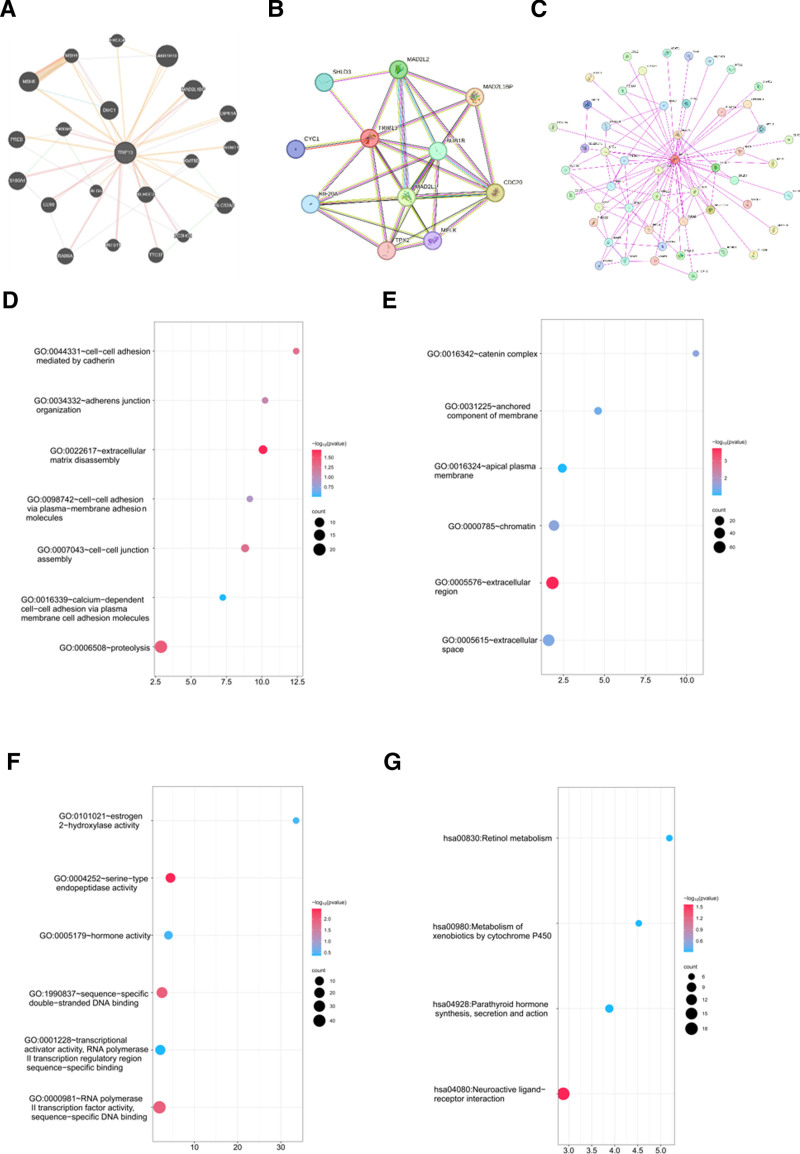
TRIP13-related gene enrichment analysis. (A) Experimentally determined TRIP13-binding genes were obtained using the GeneMANIA database. (B, C) Experimentally determined TRIP13-binding proteins were obtained using the STRING tool. (D–F) GO enrichment analysis of TRIP13-related genes. (G) KEGG pathway enrichment analysis of TRIP13-related genes.

Genes interacting with TRIP13 were identified through STRING and GeneMANIA. Additionally, numerous investigations were conducted to analyze pathway enrichment. Figure 7A shows the top 20 genes that exhibited a correlation with TRIP13 differential expression. TRIP13-binding proteins have been identified via experimental data obtained using the STRING program. TRIP13 interacted with KIF20A, TPX2, MAD2L1, MELK, BUB1B, CDC20, MAD2L1BP, MAD2L2, and SHLD3 (Fig. 7B and C).

Using data from the TCGA database, we also discovered TRIP13-related genes expressed differently in LIHC. However, only protein-coding genes with a rank of *P* < .05 and |fold change|>1 were considered. We selected 600 genes with the lowest values. The *P*-value for the enrichment of this gene set in the GO and KEGG pathways was determined. Analysis using the Gene Ontology Biological Process (GO-BP) database showed that the majority of these genes were involved in various cellular processes, including cadherin-mediated cell–cell adhesion, extracellular matrix disassembly, cell–cell adhesion via plasma membrane adhesion molecules, cell–cell junction assembly, calcium-dependent cell–cell adhesion via plasma membrane cell adhesion molecules, and proteolysis (Fig. 7D). The GO-CC analysis indicated that the majority of these genes were linked to the catenin complex, a component of the firmly attached cell membrane, as well as the apical plasma membrane, chromatin, extracellular region, and extracellular space (Fig. 7E). An analysis performed using the Gene Ontology Molecular Function (GO-MF) database showed that the majority of these genes were linked to various activities, such as estrogen 2-hydroxylase activity, serine-type endopeptidase activity, hormone activity, sequence-specific double-stranded DNA binding, transcriptional activator activity, RNA polymerase Ⅱ transcription regulatory region sequence-specific binding, and RNA polymerase Ⅱ transcription factor activity, as shown in Figure 7F. The KEGG enrichment analysis results indicated that TRIP13 was involved in various biological processes, including retinol metabolism, metabolism of xenobiotics by cytochrome p450, parathyroid hormone synthesis, secretion, and action, and neuroactive ligand–receptor interactions (Fig. 7G).

In addition, the GSEA approach was used to detect the functional enrichment patterns linked to both low and high levels of TRIP13 expression in LIHC. The main pathways involved in TRIP13 inhibition, as depicted in Figure S2, Supplemental Digital Content, https://links.lww.com/MD/P87, include complement and coagulation cascades, fatty acid metabolism, peroxisome, drug metabolism cytochrome p450, retinol metabolism, metabolism of xenobiotics by cytochrome, valine leucine, and isoleucine degradation, primary bile acid biosynthesis, Ppar signaling pathway, glycine serine and threonine metabolism, propionate metabolism, steroid hormone biosynthesis, renin angiotensin system, drug metabolism other enzymes, adipocytokine signaling pathway, arginine and proline metabolism, and linoleic acid metabolism. Concurrently, elevated levels of TRIP13 stimulate the cell cycle, DNA replication, and homologous recombination processes. Our findings suggest that TRIP13 plays a critical role in regulating the cell cycle, metabolism, and immune response, which ultimately contributes to a worse prognosis in cancer patients.

### 3.7. Establishment of a prognosis based model for TRIP13 expression in LIHC

**Figure 8.**
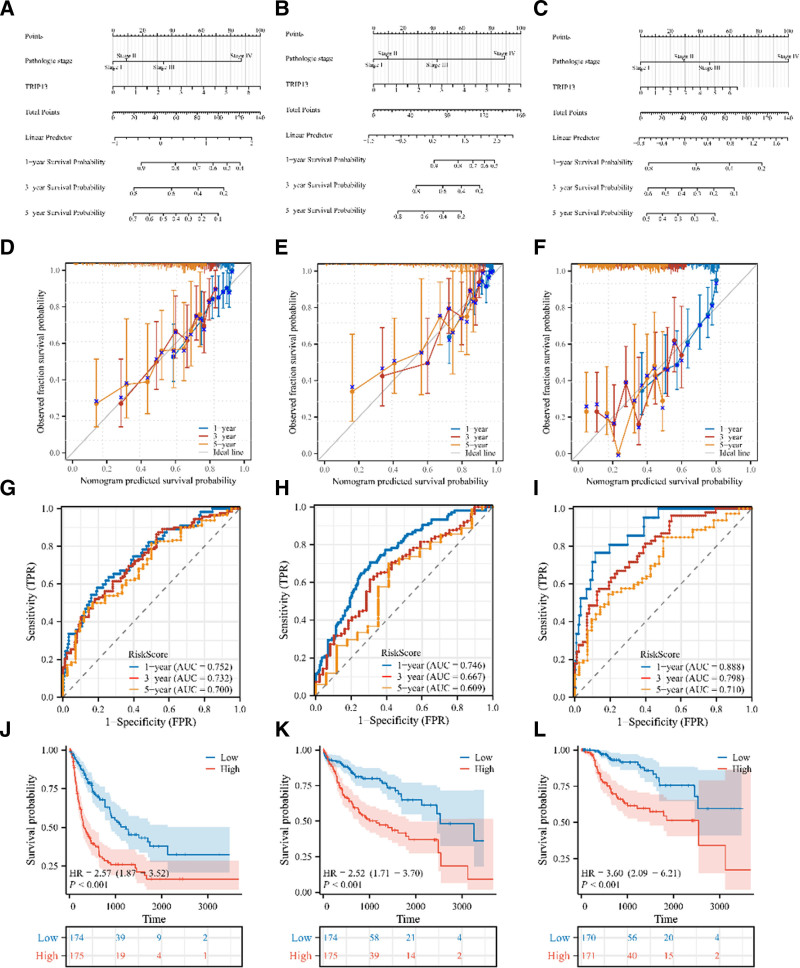
Construction and evaluation of nomogram. (A–C) Nomograms predict 1-, 3-, and 5-year OS, DSS, and PFI probabilities in LIHC patients. (D–F) Calibration curves of nomogram predictions for 1-, 3-, and 5-year OS, DSS, and PFI in patients with LIHC. (G–I) Time-dependent ROC curve analyses of a nomogram. (J–L) Kaplan–Meier analysis and corresponding ROC curves for patients with a high and low nomorisk in the TCGA-LIH cohort.

Three nomograms were developed to forecast the OS, DSS, and PFI of patients with LIHC (Fig. 8A–C). The forecasts and observations showed a high level of agreement, as seen by the bias-corrected line of the calibration plot aligning closely with the ideal curve (45° line) (Fig. 8D–F). The AUCs for the 1-, 3-, and 5-year survival rates of the constructed nomogram were > 0.6, indicating that the model exhibited a strong predictive performance (Fig. 8G–I). Furthermore, samples were categorized into high-risk and low-risk groups according to the risk index of the Norman diagram. The survival rates of the 2 patient groups were compared using the log-rank test. The Kaplan–Meier curve showed that the low-risk group had a significantly higher survival rate (*P* < .001) (Fig. 8J–L). The findings validate the fact that the Norman plot demonstrates strong predictive capability for OS, DSS, and PFS in patients with LIHC. This suggests that the Norman plot could be valuable in assessing the prognosis of LIHC patients.

### 3.8. Prediction of miRNA targets of TRIP13

**Figure 9.**
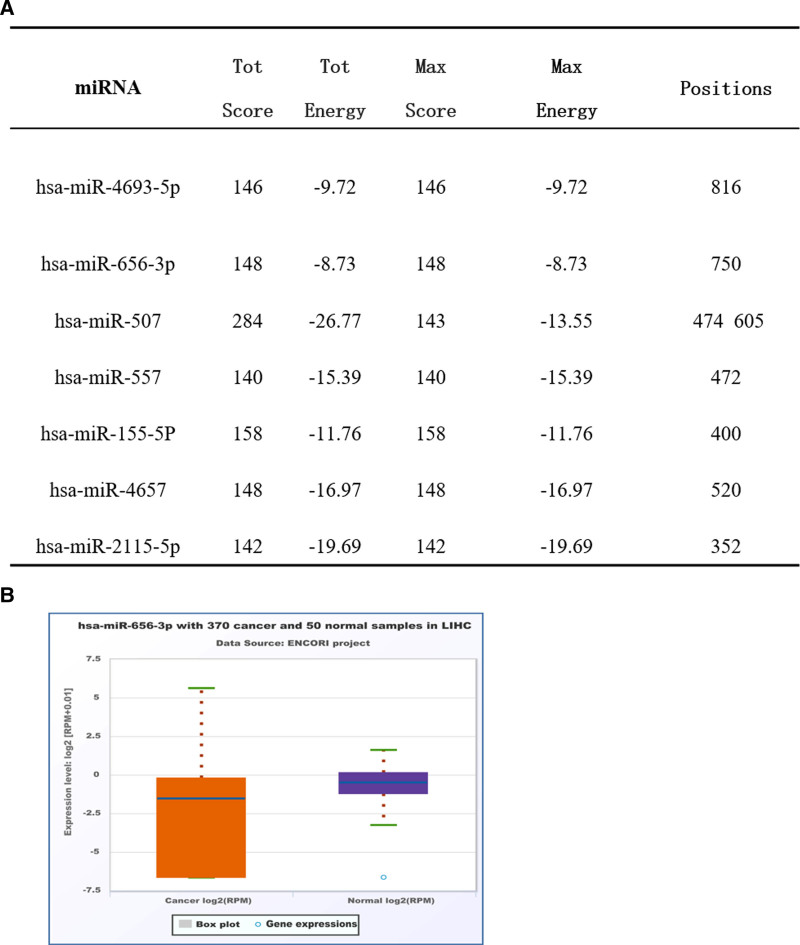
Pan-cancer correlation analysis between the TRIP13 expression level and drug sensitivity. (A) Venn plot identifying 26 overlapping predicting drugs among the CTRP and GDSC databases. (B) The relationship between TRIP13 expression levels and sensitivities of the predicting drugs.

In this investigation, we utilized the miRDB database to predict miRNAs that could potentially interact with TRIP13, and 10 miRNAs with the highest prediction scores were identified. Subsequently, the miRanda algorithm was employed to predict the site of binding of miRNA to TRIP13. Seven of these were characterized as having potential binding sites on TRIP13 (Fig. 9A). miRNA can inhibit mRNA translation by destroying its stability. Our study employed the UALCAN database to examine the expression of these 7 miRNAs in LIHC. As depicted in Figure 9B, the expression level of hsa-miR-656-3p was down-regulated (*P* = .0006), which is in contrast to the elevated expression level of TRIP13 in LIHC.

### 3.9. The correlation between TRIP13 expression and drug sensitivity

**Figure 10.**
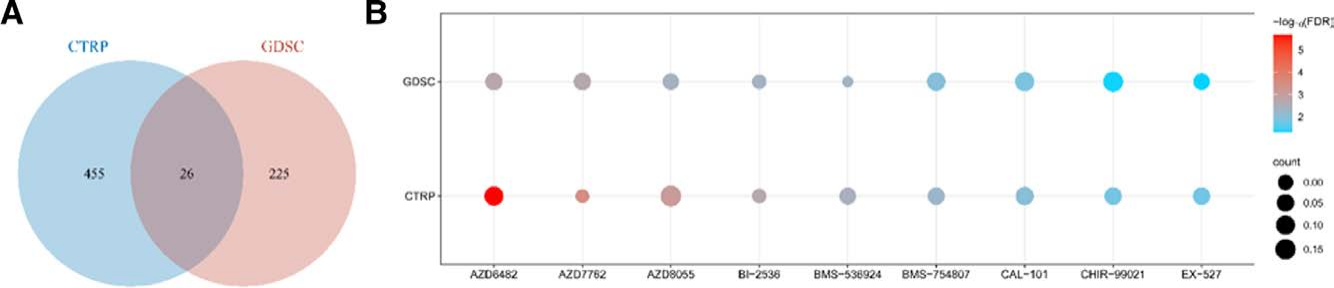
Prediction of miRNA targets of TRIP13. (A) Based on the miRDB and miRanda analyses, we detected potential targets of the combination of miRNA and TRIP13. (B) The expression levels of hsa-miR-656-3p between healthy and primary tissues in LIHC using the UALCAN dataset.

The above data suggest that TRIP13 may play a role in cancer formation. Subsequently, this investigation examined the correlation between the TRIP13 expression level and drug sensitivity using the CTRP and GDSC datasets. This study analyzed the prediction outcomes obtained with the 2 databases based on criteria such as *P* < .05 and |correlation coefficient|>0.1. The CTRP database listed 481 predicted pharmaceuticals, while the GDSC database featured 251 anticipated drugs. Using a Venn diagram (Fig. 10A), we successfully identified a total of 26 genes that were common to both cohorts. We also analyzed TRIP13 expression levels in each tumor type and evaluated the pharmacological sensitivity of these 26 drugs. Studies have shown that there is a direct relationship between TRIP13 expression levels and sensitivity to AZD6482, AZD7762, AZD8055, BI-2536, BMS-536924, BMS-754807, CAL-101, CHIR-99021, and EX-527 (Fig. 10B).

### 3.10. TRIP13 promotes lung cancer cell proliferation and metastasis

**Figure 11.**
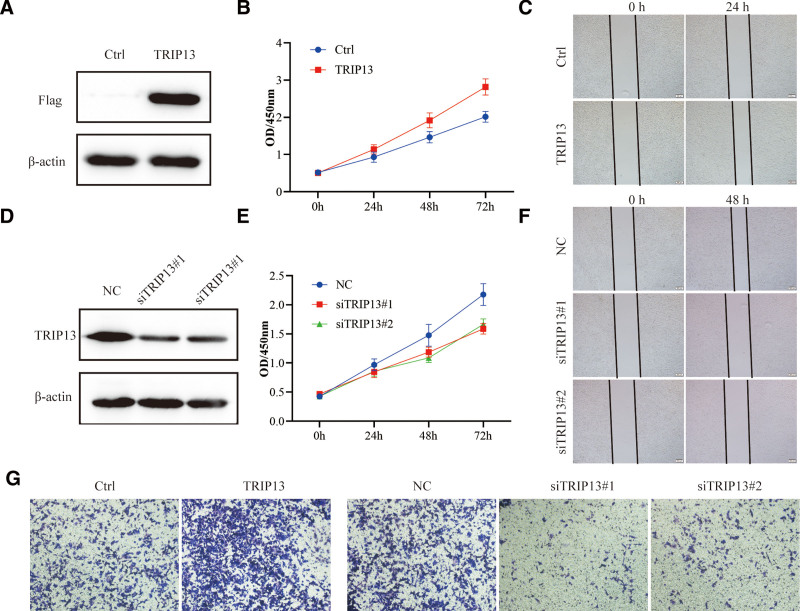
TRIP13 promotes A549 cell proliferation, migration, and invasion. A549 cells were infected with lentivirus to overexpress TRIP13, 48 hours later, (A) western blot was performed to detect the overexpression of TRIP13 using an anti-Flag antibody, (B) and cell proliferation was evaluated through CCK8, (C) the cells migration was determined by wound healing. A549 cells were transfected with siRNA specifically to TRIP13 or NC, (D) the interference efficiency was detected by western blot, (E) CCK8 was performed to detect the cell proliferative ability, (F) wound healing was performed to evaluate the cell migration. (G) The capacities of A549 cells to invade were examined by transwell assay.

To verify the impact of TRIP13 on lung cancer, A549 cells were infected with a TRIP13 overexpression lentiviral, and the cell proliferation was evaluated via CCK8 assay, the results indicated that TRIP13 was overexpressed successfully (Fig. 11A), and cell proliferation was significantly facilitated compared with the control (Fig. 11B). Wound healing assay demonstrated that overexpress TRIP13, the cells migration ability is significantly enhanced (Fig. 11C). Conversely, when the expression of TRIP13 was downregulated by siRNA (Fig. 11D), the cell proliferative ability decreased significantly (Fig. 11E), and the cell’s migration ability was inhibited (Fig. 11F). Transwell assay was performed to evaluate the cell invasion during regulating TRIP13 expression. As shown in Figure 11G, overexpression of TRIP13 significantly accelerated cell invasion, while knockdown of TRIP13 expression through transfection with specific siRNA, led to obvious suppression of cell invasion capabilities.

## 4. Discussion

*Trip13* is comprised of 14 exons that code for a protein with 432 amino acid residues.^[7]^ Trip13 protein is an AAA protein belonging to a large AAA + protein superfamily involved in numerous cellular processes, including checkpoint signaling, DNA break repair and recombination, and chromosome synapsis.^[8]^ The oncogenic roles of TRIP13 have recently drawn substantial attention. Emerging evidence has shown that TRIP13 may serve as an oncogene and could be correlated with poor survival in patients with hepatocellular carcinoma,^[9]^ breast cancer,^[10]^ and clear cell renal cell carcinoma (ccRCC).^[11]^ Pan-cancer analysis, which compares differences between various tumors, is crucial for identifying new biomarkers and therapeutic targets for tumors. In this study, the expression of TRIP13 was analyzed in multiple types of tumors. TRIP13 was significantly upregulated in BLCA, CHOL, COAD, ESCA, HNSC, LUAD, LUSC, PRAD, READ, STAD, and THCA but not in hepatocellular carcinoma, breast cancer, and clear cell renal cell carcinoma. We further explored the relationship between TRIP13 overexpression and prognosis. Survival association analysis, including an analysis of the OS, DSS, and PFI was performed using Kaplan–Meier survival curves for each type of cancer. Upon combining these results, we found that high TRIP13 expression levels were associated with a poor prognosis in ACC, KIRP, LIHC, LGG, MESO, LUAD, KIRC, and SKCM.

Growing evidence indicates that genomic mutations drive various cancers. For example, *BRCA1* and *BRCA2* mutations are significantly associated with patient survival.^[12]^ In this study, we observed that genetic amplification was the most frequent genomic alteration observed in *TRIP53*, and was first identified in LUSC. TRIP13 mutations occurred most commonly in LUSC, followed by ACC, ESCA, LUAD, and BLCA, with mutation rates above 10%. Recent studies found that TMB and MSI could be used as biomarkers for a checkpoint blockade response to several tumors; tumors with high TMB and MSI exhibited a better response to immunotherapy.^[13,14]^ The correlation between TRIP13, TMB, and MSI was examined using Spearman correlation analysis. TRIP13 expression was significantly negatively correlated with TMB in 18 tumors and with MSI in 8 tumors. Aberrant DNA methylation has been frequently observed in cancer and can accelerate tumor development by regulating cell proliferation.^[15]^ Here, differences in methylation patterns were explored using the GCSA database. A significant negative correlation was observed between TRIP13 expression levels and DNA methylation in BLCA, BRCA, ESCA, HNSC, KIRC, LIHC, LUAD, LUSC, PAAD, PRAD, and UCEC. Lower methylation levels are associated with poor outcomes.

Immune cells interact extensively with cancer cells and have been demonstrated to play an essential role in regulating cancer progression, metastasis, recurrence, and immunotherapy response.^[16]^ Various genes can influence immune cell infiltration. In this study, the immune cell infiltration, stromal score, immune score, and microenvironment score were calculated. We found that TRIP13 was negatively correlated with poor immune infiltration, especially with CD4 and CD8, in most types of cancers. Previous studies have demonstrated that the pharmacologic targeting or silencing of TRIP13 results in the suppression of the Wnt/β-catenin signaling pathway.^[17]^ Wnt/β-catenin activation was frequently associated with poor spontaneous T-cell infiltration across most cancers.^[18]^ We postulated that in addition to directly affecting tumor cells, TRIP13 could also have an immediate effect on the function of immune cells via the activation of Wnt/β-catenin. MDSCs are considered key promoters of cancer development within the tumor TME that sustain cancer progression. We found TRIP13 was negatively correlated with MDSC levels, further indicating the immunosuppressive role of TRIP13.

Using STRING and GeneMANIA databases, we identified several proteins, including KIF20A, TPX2, MAD2L1, MELK, BUB1B, CDC20, MAD2L1BP, MAD2L2, SHLD3, which were involved in interactions with TRIP13. Most of these proteins are well-characterized and play a role in cell cycle regulation. These findings corroborated the results of our gene enrichment analysis and were in accordance with those outlined in other publications, which showed that TRIP13 regulates the proliferation and cell cycle in cancer cells.^[19,20]^ To explore the clinical usefulness of the TRIP13 expression signature, we created a predictive nomogram that integrated the LIHC patients in the TCGA cohort. The nomogram demonstrated smaller prediction errors for OS, DSS, and PFS within 5 years. Using the log-rank test, this nomogram accurately predicted the OS, DSS, and PFS in the LIHC cohort (*P* < .001). This validated the prognostic predictive value of the nomogram for LIHC patients. Using the CTRP and GDSC databases, this study helped identify 9 medicines whose cellular sensitivity was positively linked with TRIP13 expression. The majority of these medications focus on preventing tumor formation and proliferation.^[21–26]^

We also investigated the upstream miRNA that regulated TRIP13 expression in this study. Using the miRDB database, we discovered 10 miRNAs that might potentially interact with TRIP13, and binding sites were identified using the miRanda algorithm. Subsequently, the UALCAN database was used to assess whether their expression levels were correlated with TRIP13 expression levels. Among these, hsa-miR-656-3p was negatively correlated with TRIP13 expression levels and acted as a potential upstream miRNA of TRIP13. A previous study revealed that the expression of hsa-miR-656-3p was down-regulated in LIHC. The overexpression of miR-656-3p suppresses the invasion, migration, and proliferation of hepatocellular carcinoma (HCC) cells.^[27]^ However, we still need to verify whether the suppressive effect of hsa-miR-656-3p on HCC resulted from the suppression of TRIP13. Additionally, in vitro experiments also demonstrated that overexpression of TRIP13 promote lung cancer cell proliferation, migration, and invasion. Conversely, the proliferation, migration, and invasion abilities of lung cancer cells are significantly reduced when the expression of TRIP13 is inhibited. In summary, our results suggested that TRIP13 was a promising prognostic biomarker and a potential predictor of sensitivity to immunotherapy in several malignant tumors and LIHC.

## Author contributions

**Conceptualization:** Chong Li, Ziyu Zhou, Lianlian Wu.

**Funding acquisition:** Chong Li, Jing Liu.

**Investigation:** Jie Teng.

**Software:** Yao Guo.

**Validation:** Chong Li, Piao Chen, Dandan Qiao.

**Writing – original draft:** Ziyu Zhou, Tingjun Liu, Quangang Chen.

**Writing – review & editing:** Yi Liu, Jing Liu.

## Supplementary Material


