# Supplementary material for: Pan-cancer analysis shows that TRIP13 as a potential prognostic and immunotherapeutic biomarker for multiple cancer types including LIHC and LUAD
Source: Medicine (Baltimore). 2025 May 30;104(22):e42588. doi: 10.1097/MD.0000000000042588 (PMC12129506; doi:10.1097/MD.0000000000042588)

Figure S1. Correlation analysis between TRIP13 expression levels and immune infiltration of MDSCs.

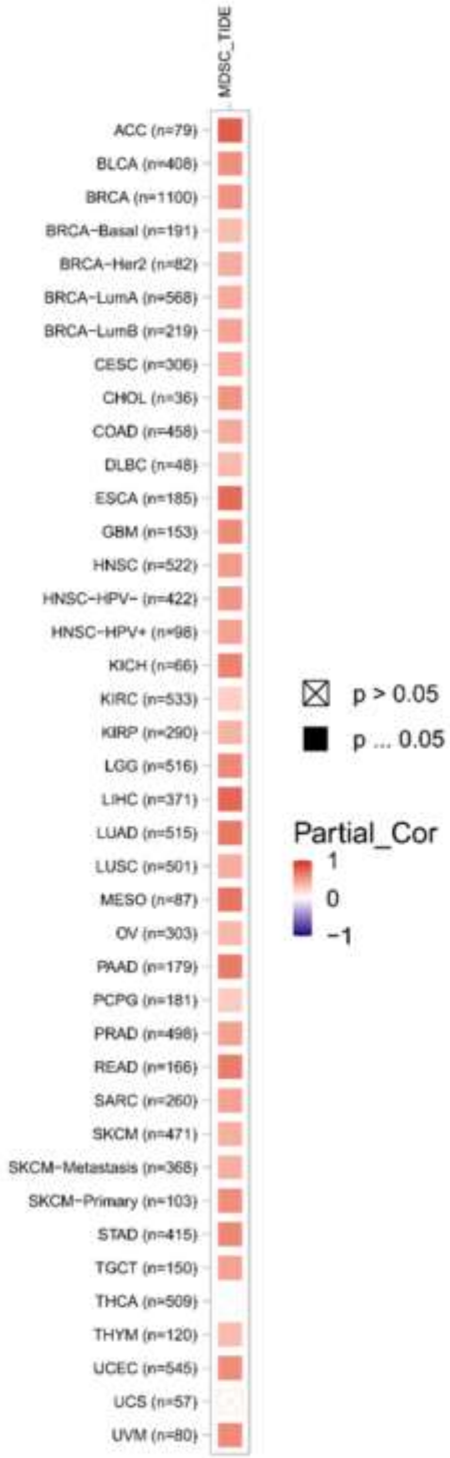

Figure S2. Gene set enrichment analysis of TRIP13.

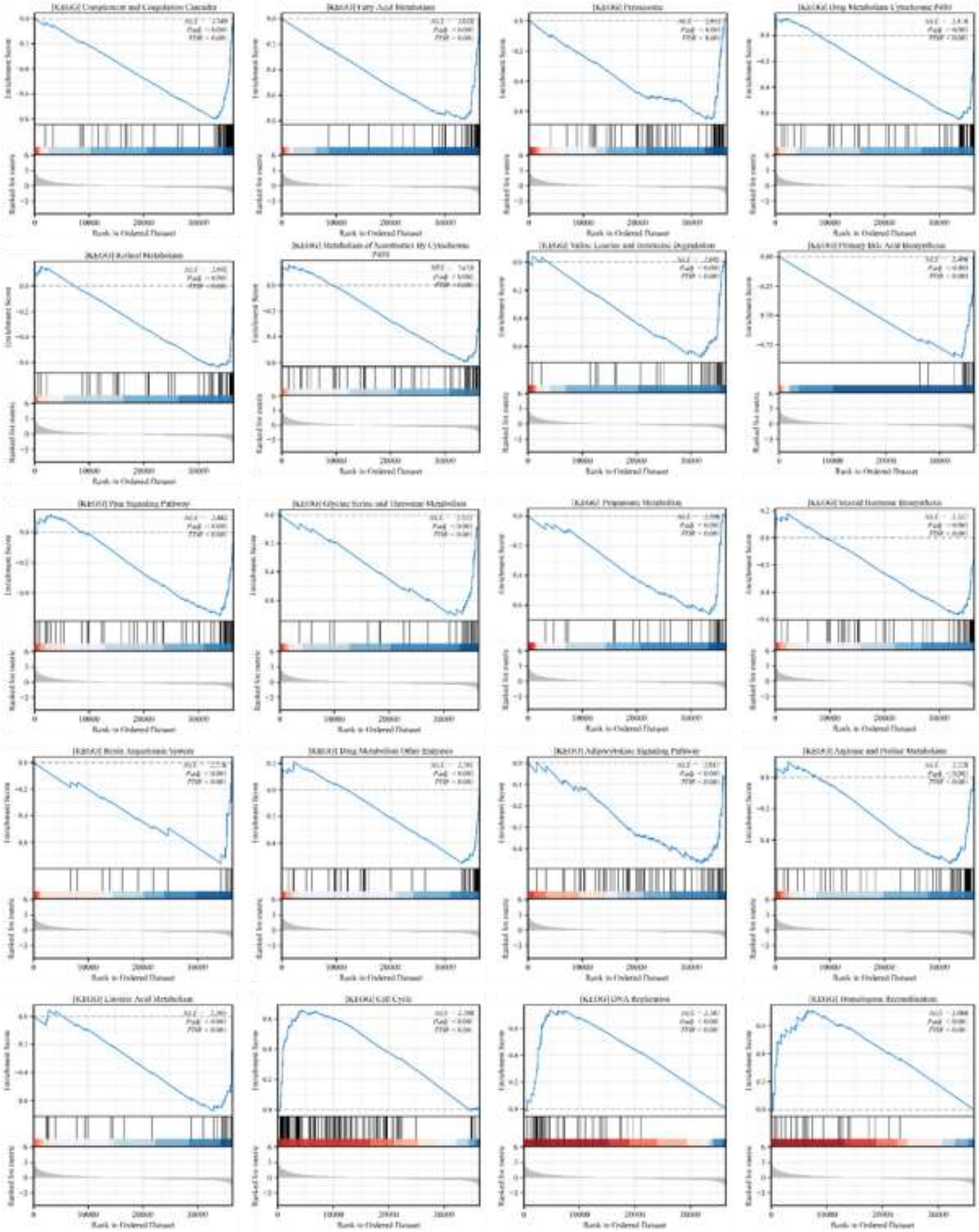

Supplement: Supplementary file 1 [file medi-104-e42588-s001.pdf]
